# Virtual Cell Based Assay simulations of intra-mitochondrial concentrations in hepatocytes and cardiomyocytes

**DOI:** 10.1016/j.tiv.2017.09.009

**Published:** 2017-12

**Authors:** Andrew P. Worth, Jochem Louisse, Peter Macko, J.V. Sala Benito, Alicia Paini

**Affiliations:** a European Commission, Joint Research Centre, Directorate F - Health, Consumers and Reference Materials, Chemical Safety and Alternative Methods Unit, EURL ECVAM, Ispra, Italy

**Keywords:** VCBA, Mitochondrial membrane potential, HepaRG, ICell cardiomyocyte, In silico, In vitro

## Abstract

In order to replace the use of animals in toxicity testing, there is a need to predict human in vivo toxic doses from concentrations that cause adverse effects in in vitro test systems. The virtual cell based assay (VCBA) has been developed to simulate intracellular concentrations as a function of time, and can be used to interpret in vitro concentration-response curves. In this study we refine and extend the VCBA model by including additional target-organ cell models and by simulating the fate and effects of chemicals at the organelle level. In particular, we describe the extension of the original VCBA to simulate chemical fate in liver (HepaRG) cells and cardiomyocytes (ICell cardiomyocytes), and we explore the effects of chemicals at the mitochondrial level. This includes a comparison of: a) in vitro results on cell viability and mitochondrial membrane potential (mmp) from two cell models (HepaRG cells and ICell cardiomyocytes); and b) VCBA simulations, including the cell and mitochondrial compartment, simulating the mmp for both cell types. This proof of concept study illustrates how the relationship between intra cellular, intra mitochondrial concentration, mmp and cell toxicity can be obtained by using the VCBA.

## Highlights

• The VCBA has been developed to simulate intracellular concentrations.
• The VCBA was extended to predict intra-mitochondrial concentrations in cardiomyocytes and HepaRG cells.
• The VCBA has potential in supporting in vitro to in vivo extrapolation.

## 1 Introduction

Numerous research initiatives are focusing on developing methods and approaches for reducing, refining, or even replacing tests on animals (Paini et al., 2010, Niklas et al., 2013, Worth et al., 2014). Among the different strategies, combinations of targeted high-throughput in vitro and in silico tools are considered to represent promising strategies for improved toxicity testing without the use of animals (Basketter et al., 2012). These new approaches could ultimately be important in evaluating the human health risk of thousands of chemicals.

In vitro models offer a high-throughput approach for assessing chemical-induced molecular and cellular changes. However, extrapolating these perturbations to an in vivo effect across chemicals, dose, time, and species is a challenge (Shah and Wambaugh, 2010). The use of silico models to describe the cellular system increases our understanding of the (adverse) effects observed in in vitro systems, and should also improve the translation of in vitro data to the in vivo situation. Since the mitochondrion is an important organelle in many toxicity pathways, the prediction of chemical effects on mitochondria is of high interest. Mitochondria perform two critical functions in the cell, namely the production of more than 90% of the cell's energy, and the control of cell survival as an integral part of programmed cell death (apoptosis) (Passarella et al., 2003).

There are three potentially adverse effects that result from mitochondrial disruption: 1. disrupted energy metabolism; 2. increased free radical generation; and 3. altered apoptosis. Here we address the disruption of mitochondrial energy metabolism as measured by changes in the mitochondrial membrane potential (mmp). The measurement of mmp provides information on the ability to couple electron transfer with ATP synthesis, as well as the ability of the organelle to take up and release ions and substrates across the inner mitochondrial membrane. It is well known that chemicals that act as inhibitors of mitochondrial ETC complexes or as uncouplers of oxidative phosphorylation can induce cell toxicity and death. Mitochondrial dysfunction triggered by inhibition of mitochondrial respiration or uncoupling of oxidative phosphorylation results in decreased ATP levels that are linked in a causative manner to the following events observed at the cellular level: (a) the loss of mitochondrial membrane potential; (b) the loss of mitochondrial protein import and protein biosynthesis; (c) reduced activities of enzymes of the mitochondrial respiratory chain and the Krebs cycle; (d) elevated levels of reactive oxygen species (ROS); (e) the loss of mitochondrial motility, causing a failure of mitochondria to re-localize to sites of increased energy demands, such as synapses; (f) destruction of the mitochondrial network; (g) increased mitochondrial uptake of Ca, causing Ca overload (Graier et al., 2007); and (h) rupture of the inner and outer mitochondrial membranes, leading to release of mitochondrial pro-death factors, including cytochrome *c*, apoptosis-inducing factor and endonuclease (Braun, 2012, Martin, 2011, Correia et al., 2012, Cozzolino et al., 2013).

**Fig. 1:**
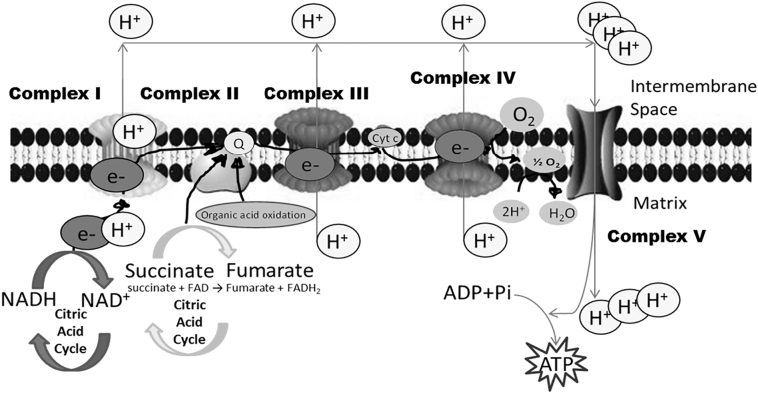
Schematic representation of the mitochondrial electron transport chain (ETC). Mitochondria generate ATP by using the energy produced through the transfer of electrons (e-) in the ETC in a mechanism that pumps protons (H +) from the matrix into the intermembrane space, creating a transmembrane potential. The NADH and succinate generated in the citric acid cycle are oxidized, providing energy to power ATP synthase (Complex V). Complex I accepts electrons from the electron carrier nicotinamide adenine dinucleotide (NADH), and passes them to coenzyme ubiquinone (Q), which also receives electrons from complex II. Q passes electrons to complex III, which passes them to cytochrome *c* (cyt c). Cyt c passes electrons to Complex IV, which uses the electrons and hydrogen ions to reduce molecular oxygen to water (picture made using Protein Lounge, www.proteinlounge.com, and adapted version features also in EFSA, 2017).

Mitochondria generate ATP by utilizing the energy produced through the transfer of electrons down the electron transport chain (ETC, Fig. 1) in a mechanism that pumps protons from the mitochondrial matrix into the intermembrane space, creating an electrochemical proton gradient (or membrane potential) across the inner mitochondrial membrane (IMM) denoted ΔΨ. The reductive transfer of electrons through ETC protein complexes I–IV in the inner mitochondria membrane provides the energy to drive protons against their concentration gradient across the inner mitochondrial membrane (out of the mitochondrial cytoplasm). This results in a net accumulation of protons (H^+^ ions) outside the membrane, which then flow back into the mitochondria through the ATP-generating F_1_/F_0_ ATP-synthase (Complex V), thus producing ATP and completing the ETC. The total force driving protons into the mitochondria (i.e., Δp), is a combination of both the mitochondrial membrane potential (Δψ_m_, a charge or electrical gradient) and the mitochondrial pH gradient (ΔpH_m_, an H^+^ chemical or concentration gradient), (Perry et al., 2011). In the presence of an exogenous chemical, this membrane potential may be altered (Nicholls and Ward, 2000, Mitchell and Moyle, 1969).

The Virtual Cell Based Assay (VCBA; Zaldívar Comenges et al., 2010, Zaldívar Comenges et al., 2011) was developed as an in silico predictive tool to simulate the fate and effects of chemicals within the well of a multi-well plate, as a function of time and under defined experimental conditions. The VCBA model (Zaldívar Comenges et al., 2016 present issue) is a mathematical model which takes into account the fate of a compound in the in vitro system, that is the partitioning between (i) the plastic wall, (ii) headspace, (iii) serum proteins, (iv) lipids, and potentially the compound dynamics within the cell. The VCBA consists also of a growth model with the cell growth phases (G1, S, G2, M phases). An additional feature takes into account the partitioning of compounds within the cell, and a toxicity model. The latter part of the model is based on two parameters: the no-effect concentration (NEC) and the killing rate (kr), linked to experimental cell viability. The main simulated property is the intracellular concentration of a specific chemical within the cell, and its corresponding effect on cell viability (Zaldívar Comenges et al., 2010, Zaldívar Comenges et al., 2011).

In the present study a mathematical description of the mitochondrion was added to the original VCBA model following the Horobin approach (Horobin et al., 2013, Horobin, 2015). By extending the VCBA to include the mitochondrial compartment, the model allows prediction of the concentration in the mitochondria, and to fit mmp experimental results.

In this paper we describe the following extensions to the original VCBA (Zaldívar Comenges et al., 2016 in press):

1. Extension to two cell models, one representing the liver (HepaRG) and one the heart (ICell cardiomyocytes). Adding these cell lines to the established VCBA is a step toward the characterization of chemical toxicity in multiple cell lines, representing different target organs and different toxic effects.
2. Addition of a mitochondrial compartment. This was done to simulate the intra-mitochondrial concentration. To predict the passage from the cell into the mitochondria, the Horobin et al., method was applied (Horobin et al., 2013, Horobin, 2015). The extent to which a molecule interacts with subcellular components, such as mitochondria, is based on the physicochemical properties (pKa, z, LogP) of the molecule (Horobin et al., 2007). Horobin and coworkers published a workflow on how to apply their approach for drug design: a physicochemical classification, a quantitative structure-activity relation (QSAR) model for low molecular weight compounds known to selectively accumulate in mitochondria, and the Fick – Nernst –Planck physicochemical model (Trapp and Horobin, 2005, Trapp et al., 2008).

These VCBA extensions are illustrated for three chemicals: carbonyl cyanide-4-(trifluoromethoxy)phenylhydrazone (FCCP), amiodarone and caffeine. Amiodarone and caffeine were selected respectively as a drug and a cosmetic ingredient affecting heart and liver cells, whereas FCCP was used as a positive control for mitochondrial dysfunction.

**Fig. 2:**
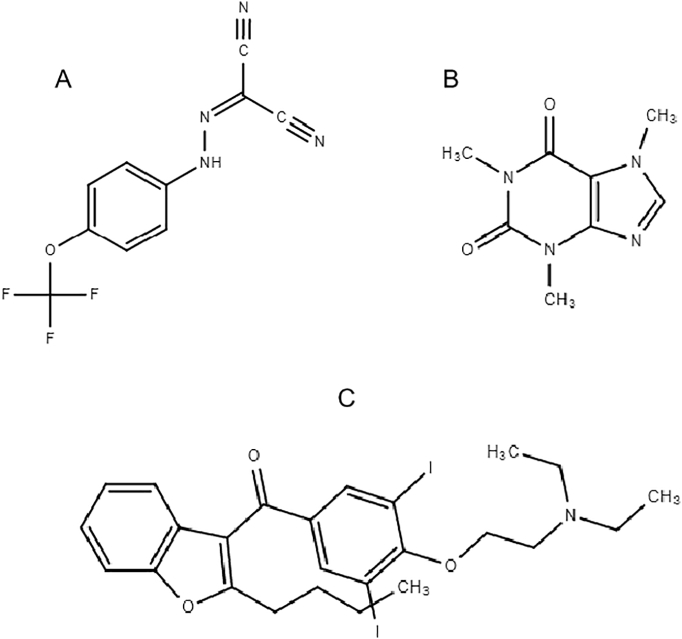
Chemical structures of FCCP (A), caffeine (B), amiodarone (C). The chemical structures were drawn using the online tool chemspider (http://www.chemspider.com/StructureSearch.aspx).

Carbonyl cyanide-4-(trifluoromethoxy)phenylhydrazone (FCCP, Fig. 2A) is a mobile ion carrier (ionophore) and is an uncoupling agent, transporting protons back to the mitochondrial matrix (Fig. 1) preventing the flow of protons through ATP synthase, thereby decreasing the mitochondrial membrane potential and the production of ATP (Heytler, 1962). It is a classic uncoupling agent because it disrupts ATP synthesis by transporting hydrogen ions through the IMM before they can be used to provide the energy for oxidative phosphorylation. It was selected as a positive chemical, known to disrupt the mmp in vitro.

Caffeine (Fig. 2B) is an alkaloid which belongs to the family of heterocyclic compounds known as purines. It is a naturally occurring stimulant found in coffee, tea, and chocolate. It is used as an additive in other beverages and is an adjuvant analgesic in some pain medications. It is also used in a wide range of cosmetic products including soaps, eye cream and cellulite cream. It is one of most widely used consumer chemicals in the world. Additionally, caffeine is one of the most commonly consumed ergogenic aids that have been shown to increase metabolic rate (Vaughan et al., 2012). Its mode of action is an increase in metabolism through binding of phosphodiesterase resulting in increased cyclic adenosine monophosphate (cAMP). Increasing cAMP activates 5′ AMP-activated protein kinase (AMPK), protein kinase A, and cAMP response element-binding causing the transcription of genes associated with increased lipid oxidation. AMPK induced by caffeine is by uncoupling electron transport resulting in an increased AMP:ATP ratio (Vaughan et al., 2012).

Amiodarone (Fig. 2C) is a class III antiarrhythmic agent. It is also a known mitochondrial toxin consisting of a benzofuran ring coupled to a p-OH-benzene structure substituted with 2 iodines and a diethyl-ethanolamine side chain. Amiodarone is extensively metabolized in the liver by cytochrome P450 3A4. The major metabolite of amiodarone is desethylamiodarone (DEA), which also has antiarrhythmic properties. In the present study amiodarone was selected as a positive chemical, known to disrupt the mmp in vitro.

## 2 Methodology

### 2.1 Test compounds

The chemicals selected were tested in HepaRG cells and ICell cardiomyocytes (Table 1). Caffeine was purchased from Fluka. Amiodarone, acetaminophen, FCCP, DMSO, and hydrocortisone hemisuccinate were purchased from Sigma-Aldrich (Milan-Italy). HyClone Fetalclone III serum was purchased from Thermo Scientific (Pittsburgh, USA). William's E medium, l-glutamine, penicillin/streptomycin, trypsin-EDTA, TMRE, Toto 3, and Hoechst 33342 for high content imaging (HCI) analysis were purchased from Invitrogen (San Giuliano Milanese, Italy).

**Table 1:** Information on test chemicals selected for the present study and relative concentrations tested for the selected cell models.

Name	CAS	MW g/mol	Concentration range tested in HepaRG™	Concentration range tested in ICell®™ cardiomyocytes
Carbonyl cyanide-*p*-trifluoromethoxyphenylhydrazone (FCCP)	370-86-5	254.17	0.5–100 μM	0–50 μM
Caffeine	58-08-2	194.19	0.195–75 mM	0–100 mM
Amiodarone	1951-25-3	645.32	0.05–50 μM	0–100 μM

### 2.2 Cell models

The cryopreserved human cell line HepaRG™ was obtained from INSERM's laboratory U522 and a cell culture bank was established in-house at the Joint Research Centre (JRC). The concentrations of test compounds tested in HepaRG™ cells are given in Table 1.

Studies were performed using ICell®™ cardiomyocytes obtained from Cellular Dynamics International (CDI). These are cardiomyocytes derived from human induced pluripotent stem cells, consisting of a mixture of spontaneously electrically active atrial, nodal, and ventricular-like myocytes. Table 1 reports the concentrations of the test compounds tested in these cells.

### 2.3 Viability and mitochondrial membrane potential (mmp) in HepaRG cells

An in house experiment using HepaRG cells exposed to different nominal concentrations (Table SM 2, in supplementary material) of the selected chemicals for 24 h was carried out. Briefly, HepaRG cells were seeded at a density of 2.6 × 10^4^ cells/cm^2^ in a growth medium composed of Williams E medium supplemented with 10% FCS, 100 units/mL penicillin, 100 μg/mL streptomycin, 5 μg/mL insulin, 2 mM glutamine and 5 × 10^− 5^ M hydrocortisone hemisuccinate. Further culturing was carried out for 2 more weeks with the same medium supplemented with 2% DMSO in 75 cm^2^ culture flask. The medium was renewed every 2 to 3 days. After differentiation, HepaRG cells were detached by gentle trypsinization, and then seeded at a density of 4–5 × 10^4^/well in 96 well microtiter plates (BD Biosciences) to allow the selection of hepatocyte-like populations. The cells were used for testing within one week after plate seeding. Compounds were diluted in culture medium with 5% HyClone Fetalclone III serum to obtain a final concentration of DMSO of 0.1%. Cells were exposed to 100 μL of exposure medium, using three wells per experimental concentration. After 24 h exposure, medium was removed and HepaRG cells were treated with a staying solution of 100 μL (10 μL TMRE, 1 μL Toto 3, and 1 μL Hoechst 33,342 in 10 mL of medium) for 30 min. This assay was performed three times (biological replicates) using, triplicate for each condition (technical replicates).

### 2.4 Viability and mitochondrial membrane potential (mmp) in ICell®™ cardiomyocytes

An in-house experiment using cardiomyocytes was carried out. Briefly, ICell®™ cardiomyocytes were cultured according to the instructions of CDI. In brief, black 96 well plates with clear bottom (Costar) were coated overnight with 0.1% gelatin (Sigma), after which 15.000 platable cells/well were plated using CDI plating medium. Cells were cultured in a humidified atmosphere, at 37 °C and with 7% CO_2_. After two days, medium was refreshed with CDI maintenance medium, and two days later, cells were ready for exposure. Compounds were diluted in CDI maintenance medium to obtain a final concentration of DMSO of 0.1%. Cells were exposed to 100 μL of the medium using three wells per experimental condition (nominal concentrations applied are reported in Table SM 2, in supplementary material). After 24 h exposure, medium was removed and cardiomyocytes were stained with TMRE, Toto 3, and Hoechst 33342 for 30 min, washed and analyzed using Cellomics, as described for the HepaRG cells. This assay was performed three times in triplicate.

### 2.5 Viability and mitochondrial membrane potential (mmp) analysis

Viability and mitochondrial membrane potential (mmp) were assessed with a high-content analysis (HCA) approach using Cellomics ArrayScan vTi (Thermo Scientific, Pittsburgh, PA, USA). A 10 × objective was used to collect 10 image fields per well for two fluorescence channels with the XF93 filter set. Cell count analysis was performed using the Target Activation Bioapplication v.4 from Cellomics Scan Software (Mennecozzi et al., 2011) on 10 taking into account around 0.8–1 × 10^4^ cells.

Mitochondrial membrane potential was determined by staining the cells with the TMRE dye (Sigma, product #87917). To this end, cells were exposed for 30 min to 100 μL of 100 μM of the TMRE dye in CDI maintenance medium, diluted from a 1000 × concentrated stock solution in nanopure water, supplemented with 10.000 × diluted Hoechst dye. Subsequently, cells were washed once with 100 μL CDI maintenance medium, after which cells were left with 50 μL CDI maintenance medium during the high content imaging studies. For the high content imaging studies, the Cellomics® ArrayScan ® VTI HCS Reader (Thermo Scientific) was used. For all analyses, the BioApplication Compartmental Analysis V4 was used. In Channel 1 the nuclei (Hoechst staining) were detected. Only normal (non-cytotoxic) nuclei were selected and in a ring of predefined size around the selected nuclei the fluorescence intensity of the dye of interest was measured (Channel 2). For the TMRE dye, the average ring spot intensity was determined and used for further calculations. This is the intensity of spots (mitochondria) normalized for the surface area in which the fluorescence was determined. The output of the high content imaging studies was used for further analyses with the total number of selected objects (live cells) and the average ring intensity per cell for TMRE.

### 2.6 The cell partitioning model including a mitochondrial compartment

**Fig. 3:**
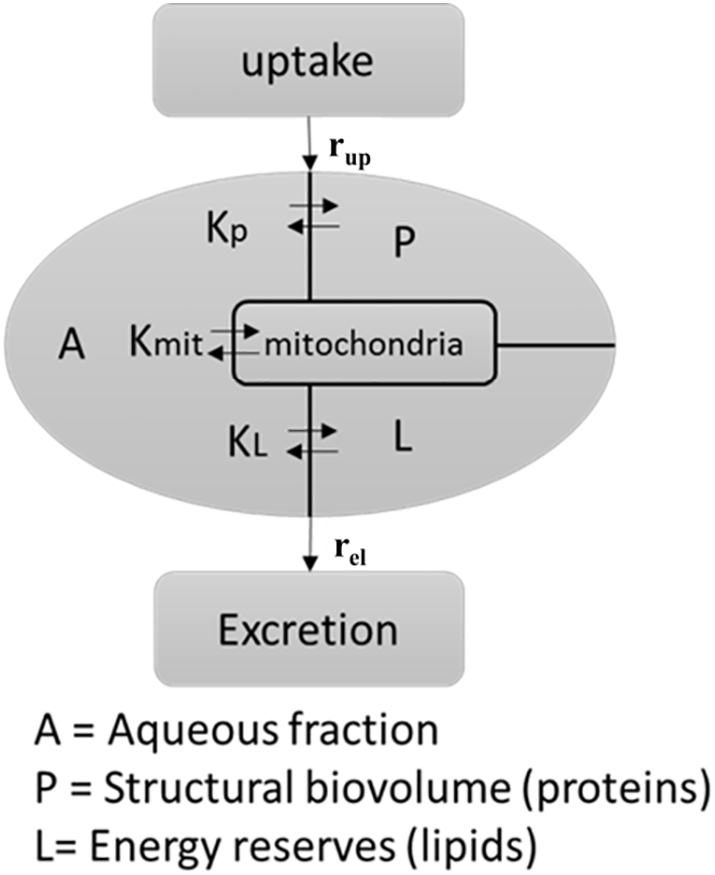
A new four-compartment cell model, including a single mitochondrial compartment.

The equations of the VCBA model are described in Zaldívar Comenges et al., 2016. The refined cell model is comprises of four compartments:- the lipid, protein, aqueous and mitochondrial compartments (Fig. 3). Mitochondria were assumed to account for 30% of the cardiomyocyte cell volume (Piquereau et al., 2013), corresponding to 10,000 mitochondria/cardiomyocyte, and 20% of the liver cell volume (Alberts et al., 2002), corresponding to 1000–2000 mitochondria/liver cell. The protein: lipid ratio in the mitochondria varies between the outer and inner membrane; the inner membrane has a ratio of 78:22, and the outer 55:45 (Guidotti, 1972). Taking into consideration the mitochondria mass fraction we can correct the cell mass fraction for its protein and lipid amounts (Table 2). For HepaRG cells, the reduction was 4% for the lipid compartment and 16% for the protein compartment; while for ICell cardiomyocytes, the reductions were 6% and 24%, respectively.

**Table 2:** Parameters of the HepaRG cells and ICell cardiomyocytes used in the virtual cell model.

Parameter type	Abbreviation used in the model	HepaRG	Cardiomyocytes	Units
Mass fraction of compartment f_x_ (aq-*aqueous*, L-lipids, P-proteins)	f_aq_	0.72	0.72	% weight
	f_L_	0.012	0.012	
	f_P_	0.268	0.268	
New Mass fraction of compartment f_x_ (aq-*aqueous*, L-lipids, P-proteins, mit-mitochondria)	f_aq_	0.72	0.72	% weight
	f_L_	0.01152	0.01128	
	f_P_	0.22512	0.20368	
	f_mit_	0.0434	0.06504	
Uptake rate	r_up_	35.208	35.208	L m^− 2^ h^− 1^
Elimination rate	r_el_	35.208	35.208	L m^− 2^ h^− 1^
Wet weight	W	1.79 × 10^− 9^	1.56 × 10^− 8^	g
Volume of the cell	V	1.67 × 10^− 15^	7.85 × 10^− 15^	m^3^
Mitochondria volume	V_mit_	3.34 × 10^− 16^ (20% of V^b^)	2.35 × 10^− 15^ (30% of V^c^)	m^3^
Cell cycle^a^ duration	G1	849	360	h
Mortality	M	1.19 × 10^− 6^	1.19 × 10^− 6^	h^− 1^

a HepaRG cells and cardiomyocytes cells do not proliferate in the assay, so the model is based on only one cell cycle step G1.

b Alberts et al., ‎2002.

c Katz, 1992

The passage of a chemical across the cell membrane occurs by a diffusion mechanism and then the chemical is distributed into the four compartments of the cell (aqueous, lipid, protein, and mitochondria compartment) (Alberts et al., 2002). The cell partitioning model was extended to explore the possibility of calculating the concentration inside the mitochondria. From the equation reported in Zaldívar Comenges et al. (2016 present issue) the total number of moles of a compound (ntot) in the cell are divided over the different compartments (Zaldívar Comenges et al., 2010, Zaldívar Comenges et al., 2011, Zaldívar Comenges et al., 2012), which now also includes the mitochondria (model equations are given in the supplementary material, while the model code can be requested from the corresponding author). More information on the equations of the neutral and dissociated forms is available in the supplementary material. From the equations in the supplementary material it is important to highlight the following to find the relation between C_mit_, C_aq_, C_b_ (with C_mit_ the concentration in the mitochondria, C_aq_ the concentration in the aqueous phase of the cell, and C_b_ the concentration in the cell) where *f*i refer to the mass fraction of each compartment (proteins, lipid, mitochondria, aqueous) in the cell and ρ_PSC_, ρ_LC_, ρ_mit_and ρ_aq_ their densities. The chemical is assumed to be in equilibrium between the different compartments (protein and lipid) with fixed values of partition coefficients in the cell K_PCell_and K_LCell_.

(1) $$C_{\mathrm{aq}}=\left(\frac{.C_{b}}{\mathrm{MW}\left(\frac{f_{\mathrm{aq}}}{\rho_{\mathrm{aq}}}+\frac{f_{l}}{\rho_{\mathrm{LC}}}K_{\mathrm{LC}ell}+\frac{f_{\mathrm{PSC}}}{\rho_{\mathrm{PSC}}}K_{PCell}+\frac{f_{m}}{\rho_{m}}K_{\mathrm{mit}}\right)}\right)$$

The chemical is assumed to enter by a diffusive mechanism from the aqueous compartment to the mitochondrial compartment. From Trapp and Horobin (2005) and Trapp et al. (2008) under steady-state conditions, the concentration ratio (K_mit_) in the cell between the aqueous phase C_aq_ and the mitochondrial C_mit_ is given by:

(2) $$K_{\mathrm{mit}}=\frac{C_{\mathrm{mit}}}{C_{\mathrm{aq}}}=\frac{{f_{n,i}}^{*}P_{n}+{f_{d,i}}^{*}{P_{d}}^{*}N/\left(e^{N}-1\right)}{{f_{n,m}}^{*}P_{n}+{f_{d,m}}^{*}{P_{d}}^{*}e^{N*}N/\left(e^{N}-1\right)}$$

A two-parameter log-logistic function was used to obtain a fit between the nominal chemical concentration and the measured mmp:

(3) $$f\left(x\right)=\frac{1}{1+\exp\left(\beta \left(\log\left(x\right)-\log\left(\alpha \right)\right)\right)}$$

where f(x) is the mmp and x is the chemical concentration. To fit the experimental data we used the function dmr with fct parameter set LL.2 from the R package drc.

### 2.7 VCBA optimization

The first step when new cell lines are introduced in the VCBA model is the optimization of the no effect concentration (NEC) and the killing rate (k_r_) for the chemical/s under study. The in vitro viability data were used to optimize the new VCBA (see Zaldívar Comenges et al., 2016, in press), to derive the no effect concentration (NEC) and the killing rate (k_r_). Optimization of both parameters is obtained by minimizing the square errors between the theoretical and experimental viability results for the different concentrations tested.

## 3 Results

### 3.1 Viability and mitochondrial membrane potential in hepatocytes and cardiomyocytes

In this section, we report the in vitro results obtained using HepaRG and ICell cardiomyocytes, for cell viability and mitochondrial membrane potential (mmp) readouts.

**Fig. 4:**
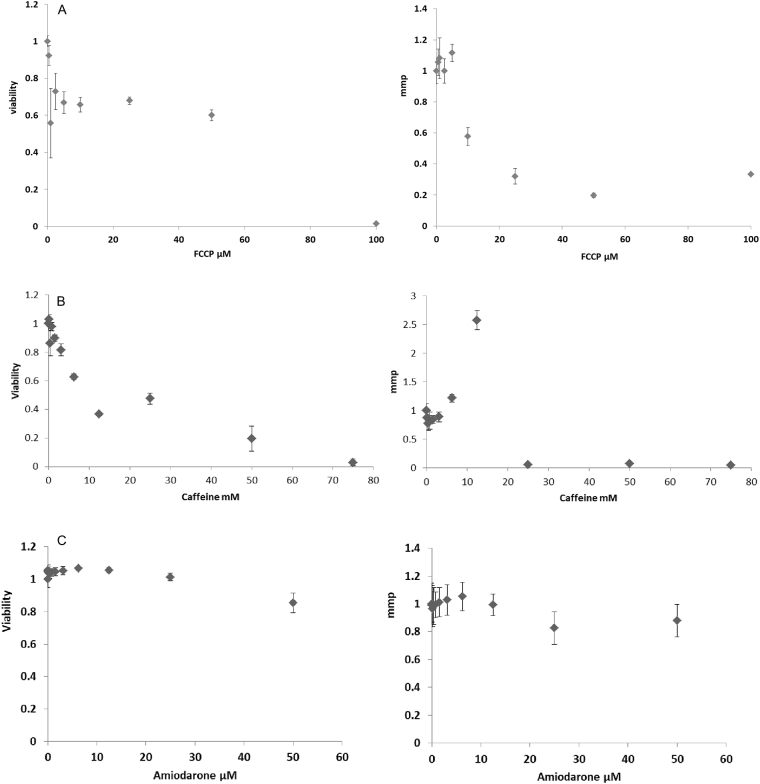
HepaRG cell viability and mmp results after 24 h exposure to different in vitro external nominal concentration of A. FCCP (μM). B. of caffeine (mM). C. of amiodarone (μM). Data are represented as % of the solvent control (100%). The Y-axis scale is normalized to the control; viability is expressed as % of surviving cells and the mitochondrial membrane potential is in Relative Fluorescence Units (RFU).

Fig. 4A shows cell viability and mmp after exposure of HepaRG cells to FFCP. Upon 24 h incubation, a slight reduction in cell viability was observed up to 50 μM whereas at 100 μM, a drastic reduction was measured. No effect on mmp was observed until 5 μM, whereas the mmp reduced to 40% at 10 μM and continued decreasing until 100 μM.

Fig. 4B shows cell viability and mmp after a 24 h exposure of HepaRG cells to caffeine. A concentration –dependent decrease of cell viability was found starting at 1.5 mM (20% loss of viable cells). A sharp increase in mmp was observed at 12.5 mM, followed by a drastic reduction at higher concentrations.

Fig. 4C shows the cell viability and mmp in HepaRG cells exposed for 24 h to amiodarone, respectively. No effect on cell viability and mmp was found at the concentrations tested.

**Fig. 5:**
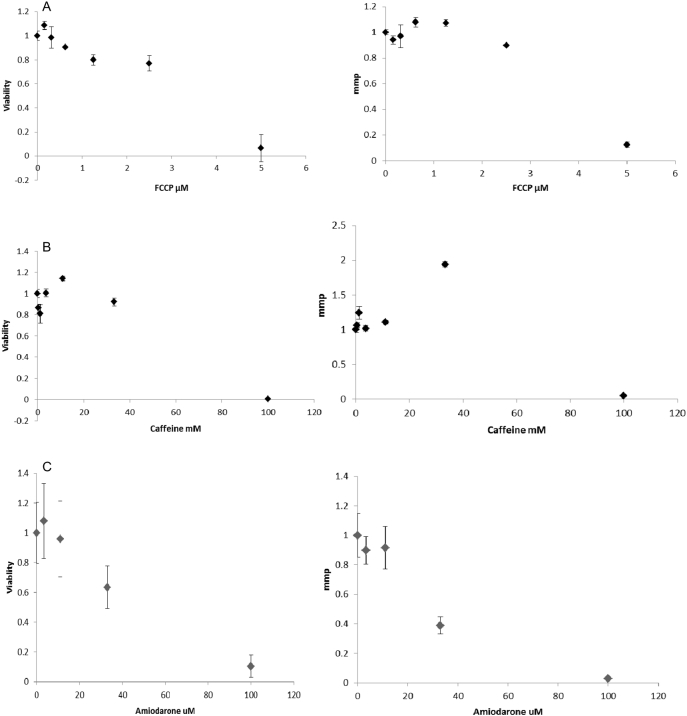
ICell Cardiomyocyte cell viability and mmp after 24 h exposure to increasing in vitro external nominal concentrations of A. FFCP (μM). B. of caffeine (mM). C. of amiodarone (μM). Data are represented as % of the solvent control (100%). Y-axis scale is normalized to the control; and for viability is expressed as % of surviving cells and the mitochondrial membrane potential is in Relative Fluorescence Units (RFU).

The effects of FCCP on cell viability and mmp in ICell cardiomyocytes are shown in Fig. 5A. A large effect can be seen for both readouts at 5 μM. Fig. 5B shows the effect of caffeine on cell viability and mmp in cardiomyocytes. After 24 h exposure, the mmp increased at 33 mM and decreased to almost zero at 100 mM, due to complete cell death. The increased signal for mmp at 33 mM was not found for the viability readout. Fig. 5C shows the effects of amiodarone on cell viability and mmp. In cardiomyocytes after exposure of 24 h the mmp and cell viability share the same decreasing slope.

### 3.2 Optimized NEC and k_r_

The first step when new cell lines are introduced in the VCBA model is the optimisation of the no effect concentration (NEC) and the killing rate (k_r_) for the chemical/s under study. Table 3 reports the optimized values. In the present optimization is done using the in vitro data for cell viability (Fig. 4, Fig. 5) for both cell lines.

**Table 3:** NEC and k_r_ used for each chemical and for both cell types.

	**FCCP**	**Caffeine**	**Amiodarone**
**HepaRG**			
NEC	0	0	0.000692
k_r_	1.611	0.222	0.199

**Cardiomyocytes**			
NEC	0	0.0046	0.00051
k_r_	242.61	0.364	1.098

### 3.3 Simulation of the intracellular and mitochondrial concentration

**Fig. 6:**
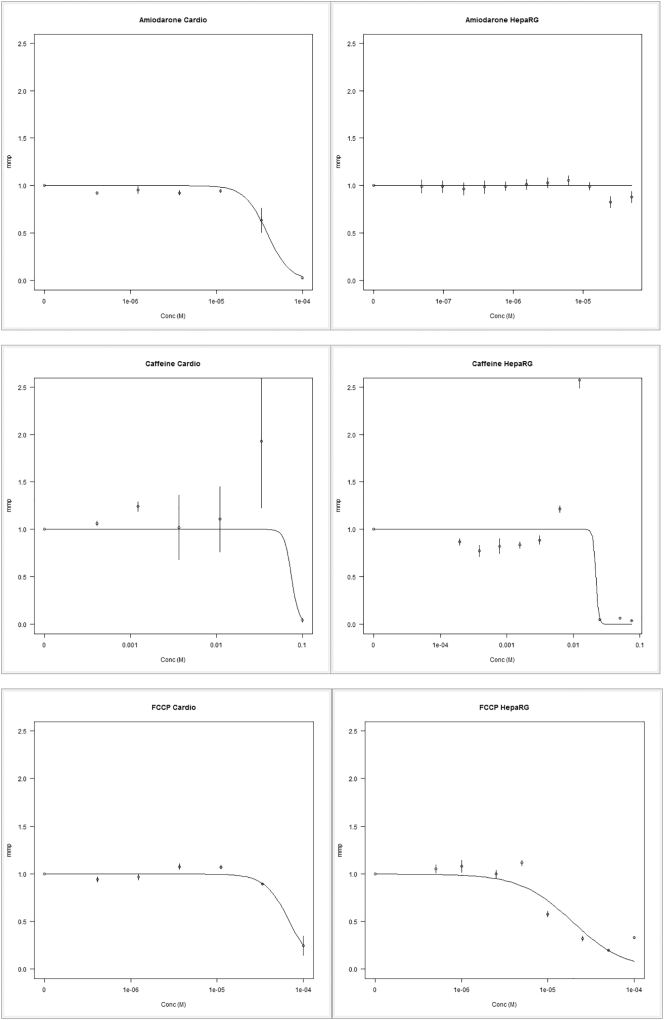
Experimental mitochondrial membrane potential data (dotted points) versus nominal concentrations (Cardio represents cardiomyocytes).

The intra-cellular and intra-mitochondrial chemical concentrations were estimated using the VCBA for the two cell lines and the three chemicals under study, using Eq. (3). Furthermore, to study the influence of chemical concentration on the mmp, a two-parameter (α,β) log-logistic model (Eq. 3, see Fig. 6) was fitted to the data. The experimental results were fitted, and the parameter α can be taken as an estimation of the IC50%; which describes the nominal chemical concentration that triggers a loss (inhibition) of 50% of the mitochondrial membrane potential. In Table 4 IC50% (α) and its respective C_b_ and C_mit_ are reported. Table 4 reports for the intracellular concentration (C_b_, g ∗ g_wetweight_^− 1^) and the intra-mitochondrial chemical concentration (C_mit_, g ∗ g_wetweight_^− 1^), for the 24 h experimental range of nominal chemical concentrations (conc. in molar M) at the corresponding IC50%. Table SM2 in supplementary material reports the simulated Cb, Caq, and Cmit for all the concentrations tested in vitro.

**Table 4:** Range of nominal concentrations (molar) for amiodarone, caffeine, and FCCP and relative amounts predicted in each of the VCBA defined compartments: intracellular (C_b_, g·g_ww_ − 1). and mitochondrion (C_mit_, g·g_ww_ − 1). For caffeine two pKa values were found based on a temperature of 40 °C (pka 10.4) and 25 °C (pKa of 14), found in pubchem. For amiodarone we found an experimental pKa value (EXP) of 6.59 (pubchem), however in most of the search engine and web based tool (such as ACD Labs) the predicted (P) pKa value of amiodarone was in a range between 8.8 and 9.4. For FCCP only one predicted value was found using ACD labs. C = concentration. α (IC50) is the concentration that gives 50% decrease in mitochondrial membrane potential.

HepaRG									
Caffeine	Nominal C (M)	Cb (g·g_ww_ − 1)	Caq (g·g_ww_ − 1)	Cmit (g·g_ww_− 1)	Caffeine	Nominal C (M)	Cb (g·g_ww_ − 1)	Caq (g·g_ww_ − 1)	Cmit (g·g_ww_ − 1)
pKa 10.4	z 1	i 1			pKa 14	z 1	i − 1		
α (IC50)	2.2E − 02	1.4E − 04	4.1E − 04	2.2E − 09		2.2E − 02	1.4E − 04	4.1E − 04	5.4E − 13


ICell Cardiomyocytes									
Caffeine	Nominal C (M)	Cb (g·g_ww_ − 1)	Caq (g·g_ww_ − 1)	Cmit (g·g_ww_ − 1)	Caffeine	Nominal C (M)	Cb (g·g_ww_ − 1)	Caq (g·g_ww_ − 1)	Cmit (g·g_ww_ − 1)
pKa 10.4	z 1	i 1			pKa 14	z 1	i − 1		
α (IC50)	7.5E − 02	5.5E − 05	1.8E − 04	9.2E − 10		7.5E − 02	5.5E − 05	1.8E − 04	2.3E − 13


HepaRG									
Caffeine	Nominal C (M)	Cb (g·g_ww_ − 1)	Caq (g·g_ww_ − 1)	Cmit (g·g_ww_ − 1)	Amiodarone	Nominal C (M)	Cb (g·g_ww_ − 1)	Caq (g·g_ww_ − 1)	Cmit (g·g_ww_ − 1)
pKa 6.56	z 0	i − 1			pKa 8.8	z 1	i − 1		
α (IC50)	6.4E − 04	2.2E − 05	2.6E − 11	1.4E − 11		6.4E − 04	1.0E − 05	1.2E − 11	3.3E − 14


ICell cardiomyocytes									
Caffeine	Nominal C (M)	Cb (g·g_ww_ − 1)	Caq (g·g_ww_ − 1)	Cmit (g·g_ww_ − 1)	Amiodarone	Nominal C (M)	Cb (g·g_ww_ − 1)	Caq (g·g_ww_ − 1)	Cmit (g·g_ww_ − 1)
pKa 6.56	z 0	i − 1			pKa 8.8	z 1	i − 1		
α (IC50)	3.9E − 05	9.5E − 08	1.1E − 13	6.3E − 14		3.9E − 05	9.5E − 08	1.2E − 13	3.2E − 16


HepaRG					ICell cardiomyocytes				
FCCP	Nominal C (M)	Cb (g·g_ww_ − 1)	Caq (g·g_ww_ − 1)	Cmit (g·g_ww_ − 1)	FCCP	Nominal C (M)	Cb (g·g_ww_ − 1)	Caq (g·g_ww_ − 1)	Cmit (g·g_ww_ − 1)
pKa 6.2	z 0	i − 1			pKa 6.2	z 0	i − 1		
α (IC50)	1.9E − 05	1.6E − 07	1.3E − 08	1.2E − 08		1.9E − 05	1.9E − 08	1.6E − 09	1.4E − 09

## 4 Discussion

In order to replace the use of animals in toxicity testing there is a need to predict human in vivo toxic doses from concentrations that cause toxicologically relevant effects in in vitro test systems. To carry out this extrapolation it is necessary to calculate target concentrations in both systems and then compare them, taking into consideration both toxicokinetics (TK) and toxicodynamics (TD). The characterization of the internal concentration that produces an effect is necessary for two applications: first, to better interpret the results of vitro experiments, since “nominal” concentrations do not represent the real concentration experienced by the cell; and, second, in extrapolating to humans, since the true concentration experienced by cells within the target organ is more relevant for human toxicity assessment. In order to address the first of these applications, we developed a Virtual Cell Based Assay (VCBA) for several cell lines (Zaldívar Comenges et al., 2016). This model takes into account the fate of a compound in the in vitro cell-based assay: partitioning between (i) the plastic wall, (ii) headspace, (iii) serum proteins, (iv) cells. The in vitro fate model is coupled with a cell growth model and a toxicodynamic effects model. Originally we developed the VCBA to predict toxicity in 3T3 balb/c fibroblast (Zaldívar Comenges et al., 2010, Zaldívar Comenges et al., 2011) and A549 Lung cells (Zaldívar Comenges et al., 2012). In order to predict additional relevant organs for toxicity testing we have now extended the VCBA to include two cell lines: HepaRG and ICell cardiomyocytes. In addition, since there is a lack of in silico methods to predict chemically induced mitochondrial dysfunction we have included in the cell model a new compartment representing mitochondria. The uptake of chemicals into the mitochondria was described using the Trapp and Horobin (2005) approach. The basic features of the VCBA were taken from the similar model developed by Armitage et al. (2014) and Kramer, 2010, Kramer et al., 2012. Kramer's model takes into account the partition of a chemical between five phases in a closed system: air, medium, protein, plastic, and cell. However, the model does not take into account cell population and cell growth. Furthermore, while metabolism is neglected in these models, the VCBA includes equations describing Michaelis-Menten kinetics, using V_max_ and K_m_. Other groups have addressed the issue of calculation of the intracellular concentration of a chemical that actually perturbs the cell (Truisi et al., 2015, Stadnicka-Michalak et al., 2014, Broeders et al., 2014, Heringa et al., 2004, Gülden et al., 2001) and more recently (Fischer et al., 2017).

The in vitro data showed differences in the behaviour of the selected chemicals. The caffeine results, for instance, show that the mmp increases before dropping rapidly. This last effect can be explained by a loss in the integrity of the outer mitochondrial membrane which can trigger the release of certain proteins mediating the cell death. The unbalanced mmp is caused by a multi-protein complex that crosses both the inner and the outer membrane forming a pore that allows the exchange of ions and other molecules smaller than 1500 Da (Waldhauser, 2008). The membrane potential cannot be sustained as a consequence of the osmotic swelling of the mitochondria, the rupturing of the outer mitochondrial membrane and the release of the death proteins. Finally, the ATP production collapses and, if enough mitochondria are affected, the cell dies (Waldhauser, 2008). The possibility of the selected chemical to trigger multiple protein complex to form channel through the membrane was not investigated further. However it would be worth investigating more concentrations to understand if such a peak registered in the mmp measurements of cells exposed to caffeine could be found for other chemicals, maybe allowing to set a biological threshold.

Using the in vitro viability measurements we optimized the VCBA for each chemical and each cell line by optimizing the NEC and k_r_ parameters. The VCBA simulation for the mmp gave a reasonable match to the in vitro data for AMI and FCCP. For caffeine the simulations were less consistent, an outlier was noticed in both cell models applied. This could be caused by an intense signal of the mitochondria trying to cope with the dysfunction, which is not captured by our model.

The virtual cell model was extended to describe a passive diffusion of chemicals from the cellular cytosol into the mitochondria using the Trapp and Horobin (2005) approach, which is based partly on pKa. The pKa of a chemical influences lipophilicity, solubility, protein binding and permeability which in turn directly affects kinetic characteristics such as absorption, distribution, metabolism and excretion. For the two bases, caffeine and amiodarone, two pKa were analyzed, for caffeine we found in literature two experimental values at two different temperatures one room temperature and one around 40 °C (which could be more close to the body as well as in vitro experimental temperatures). The different pKa values for caffeine did not impact much on the uptake in the mitochondria, since the charge of the molecule did not change. Additionally, two pKa values were found for amiodarone, one experimental (6.59) one predicted (8.8). Although the experimental value was favoured we tested both values since the predicted values are reported in many databases. The pKa values influenced the intra-mitochondrial concentration of two (2) to three (3) orders of magnitude increase with the lowest pKa (at a pKa value of 6.59 amiodarone is uncharged; and his uptake is favoured, however, with the higher pKa amiodarone becomes a protonated organic base at the experimental pH of 7.5, making the chemical less easy to uptake by the cell, this is also reflected in the predicted IC50 simulated, Table 4). This shows that the pKa - pH relationship plays a crucial role in chemical uptake. Ionisation is an important process that should not be underestimated and was recently addressed within a fate and transport model (Fischer et al., 2017).

In vitro systems are still far from able to mimic a human body and the data collected need to be interpolated and extrapolated in various ways (Bois et al., 2016). We proposed this model to help in refinement of experimental strategies, fill in knowledge gaps in adverse outcome pathways development and provide additional information to support in vitro to in vivo extrapolation (IVIVE), which is a quantitative translation of in vitro data to the in vivo organism level, for instance going from an in vitro tested concentration to a human exposure dose. Computational models can help, addressing various scales of complexity: from the sub-cellular level (mitochondrion), through the cellular one (fate and transport - VCBA like - models), the tissue and organ scale, up to the whole body (with PBK models). Future human safety assessments are likely to rely strongly on the use of multi-scale models, implemented through a combination of computational tools, in order to perform extrapolations such as IVIVE. An example has been provided for caffeine (Gajewska et al., 2015). These preliminary results should be applied in IVIVE approach for the other chemicals tested, and by including a heart compartment (to link toxicity from cardiomyocytes) in PBK modelling a refinement in organ-specific chemical toxicity could be explored. The application of the combined modelling (VCBA and PBK models) represents a step forward in improving the prediction of human toxicity and risk assessment based solely on in vitro data.

Although applied to a limited number of test chemicals, this proof of concept study illustrates how the relationship between intra cellular, intra mitochondrial concentration, mitochondrial disruption at the IC50 and cell toxicity can be obtained by using the VCBA. This approach can be used to guide the design and interpretation of in vitro experiments, and may have value in extrapolating in vitro data to the in vivo situation.

## Transparency document

Transparency document. Image 1

## Supplementary data

Supplementary material Image 2
