# Supplementary material for: Virtual Cell Based Assay simulations of intra-mitochondrial concentrations in hepatocytes and cardiomyocytes
Source: Toxicol In Vitro. 2017 Dec;45:222–32. doi: 10.1016/j.tiv.2017.09.009 (PMC5745147; doi:10.1016/j.tiv.2017.09.009)
Supplement: Supplementary file 2 — Supplementary material [file mmc2.docx]

### Supplementary material to

***Virtual Cell Based Assay simulation of intra-mitochondrial concentration in hepatocytes and cardiomyocytes***

***Andrew P Worth*** ^a^***, Jochem Louisse*** ^a^***, Peter Macko*** ^a^***, Jose Vicente Sala Benito***^a^***, Alicia Paini***^a^*

**Affiliation:**

^a^ Chemical Safety and Alternative Methods Unit, EURL ECVAM, Directorate F - Health, Consumers and Reference Materials, Joint Research Centre, European Commission, Ispra, Italy.

****Corresponding author:*** alicia.paini@ec.europa.eu

European Commission

Joint Research Centre

Directorate F - Health, Consumers and Reference Materials

Chemical Safety and Alternative Methods Unit and EURL ECVAM

Via E. Fermi 2749, TP 126

I-21027 Ispra (VA), Italy

tel.+39-0332-78 9566

fax +39-0332-78 9963

### The cell partitioning model with the mitochondrial compartment

### The cell partitioning model of the virtual cell based assay (VCBA) was extended to explore the possibility to calculate the concentration inside the mitochondria. From the equation reported in Zaldivar et al., (2016 present issue) the total number of moles of a compound (*n_tot_*) in the cell are divided over the different compartments (Zaldivar et al., 2010, 2011, 2012), which will include now also the mitochondria:

$n_{tot}=n_{aq}+n_{PSC}+n_{LC}+n_{mit}=\left(V_{aq}.C_{aq}+V_{PC}.C_{PC}+V_{LC}.C_{LC}+V_{mit}C_{mit}\right)$ 1

where the V_i_^'s^ refer to the compartment volumes (L) and the C_i_^'s^ refer to the concentration in the compartments (mol L^-1^), water, protein, lipid and mitochondria. Also the total number of moles of a chemical can be expressed as:

$n_{tot}=W.C_{b}/MW$ 2

where W is the cell weight (g), MW is the molecular weight of the chemical (g mol^-1^) and C_b_ is the chemical concentration in the cell, defined in gram on gram in wet weight (g.gww^-1^).

The chemical is assumed to be in equilibrium between the different compartments with fixed values partition coefficients:

$K_{PC}=\frac{C_{PC}/\left[PC\right]}{C_{aq}}$; 3

$K_{LC}=\frac{C_{LC}/\left[LC\right]}{C_{aq}}$; 4

The time evolution of this substance in the cell can be calculated by a simple mass balance, assuming that the uptake and elimination rates r_up_ and r_el_ (L.cm^-2^.s^-1^) are proportional to the surface area of the cell (passive diffusion) and the transfer occurs through the aqueous compartment only as:

$\frac{dn_{tot}}{dt}=V^{2/3}\times\left(r_{up}\times C_{diss}-r_{el}\times C_{b}\right)$ 5

where C_diss_ and C_aq_ refer to the chemical concentration (mol L^-1^) outside of the cell in the medium and in the aqueous compartment of the cell (mol L^-1^), respectively. Appling the chain rule of derivation to eq 2 we have:

$\frac{dn_{tot}}{dt}=\frac{1}{MW}\left(W\frac{dC_{b}}{dt}+C_{b}\frac{dW}{dt}\right)$ 6

and rearranging terms we obtain:

$\frac{dC_{b}}{dt}=\frac{MW.V^{2/3}}{W}\left(r_{up}.C_{dis}-r_{el}.C_{aq}\right)-\frac{C_{b}}{W}\frac{dW}{dt}$ 7

the last term represents the dilution due to growth of the cell. Since the concentration in the aqueous fraction C_aq_ is not a value that is measured, then we have to convert in terms of C_b_ using the partitioning approach. The wet weight, W can also be expressed as a function of the volumes of the different compartments:

$W=\rho.V=\rho\left(V_{aq}+V_{PSC}+V_{LC}+V_{mit}\right)$ 8

On the other hand:

$V_{PSC}=W_{PSC}/\rho_{PSC}$ 9

$V_{LC}=W_{LC}/\rho_{LC}$ 10

$V_{mit}=W_{mit}/\rho_{mit}$ 11

$V_{aq}=W_{aq}/\rho_{aq}$ 12

where W_PSC_, W_LC_, W_mit_, and W_aq_ are the masses of proteins, lipids, mitochondria and aqueous compartments in the cells and ρ_PSC_, ρ_LC_, ρ_mit_ and ρ_aq_ their densities.

To find the relation between C_aq_ and C_b_ we have to combine n_tot_ in eqs 1-2, the partition coefficients and eqs 9-12, then we have:

$C_{aq}=\left(\frac{.C_{b}}{MW\left(\frac{f_{aq}}{\rho_{aq}}+\frac{f_{l}}{\rho_{LC}}K_{LC}+\frac{f_{PSC}}{\rho_{PSC}}K_{PSC}+\frac{f_{m}}{\rho_{m}}K_{mit}\right)}\right)$ 13

where $f$_i_ refer to the mass fraction of each compartment (aqueous, lipid, proteins, mitochondria) in the cell. Replacing this equation into eq.8 and rearranging we obtain:

$$\frac{dC_{b}}{dt}=\left(\frac{MW.V^{\frac{2}{3}}}{W}.r_{da}\right).C_{diss}-\left(\frac{V^{2/3}}{W\left(\frac{f_{aq}}{\rho_{aq}}+\frac{f_{l}}{\rho_{LC}}K_{LC}+\frac{f_{PSC}}{\rho_{PSC}}K_{PSC}+\frac{f_{m}}{\rho_{m}}K_{mit}\right)}.r_{ad}\right).C_{b}-\left(\frac{1}{W}\frac{dW}{dt}\right).C_{b}$$

14

This equation gives the chemical concentration inside the cell as a function of time.

For estimation of the chemical partitioning inside the cell (lipid and protein) and estimation of cell permeability please see Zaldivar et al., 2016, present issue.

### We describe here the approach with regards to the mitochondrial model compartment; the chemical enters through a diffusive mechanism from the aqueous compartment to mitochondrial compartment:

$\frac{dC_{mit}}{dt}=\frac{V_{mit}^{2/3}}{W_{mit}}*(r_{up\cdot}C_{aq}-r_{el}C_{mit})$ 15

Where C_aq_ is the concentration in the aqueous phase, C_mit_ is the concentration inside the mitochondria, and we assume that the uptake and elimination rate are equal, $\cdot r_{up}=r_{el}$.

We also assume that the chemical enters into the mitochondria by rapid partitioning so that we can use the partitioning constant K_mit_:

$K_{mit}=\frac{\left|HA_{m}\right|}{\left|HA_{i}\right|}$ 16

here $\left|HA_{m}\right|$ and $\left|HA_{i}\right|$ refers the neutral form of the chemical inside of mitochondria and cell respectively. In our case $\left|HA_{i}\right|$ is calculated from the VCBA model by introducing a mitochondrial term as described in 14.

$\left|AH_{i}\right|=\frac{C_{b}}{MW.\left(\frac{f_{aq}}{\rho_{aq}}+\frac{f_{l}}{\rho_{L}}K_{L}+\frac{f_{P}}{\rho_{P}}K_{P}+\frac{f_{m}}{\rho_{m}}K_{mit}\right)}$ 17

From the Trapp & Horobin, (2005) and Trapp et al., (2008) under steady-state conditions, and the concentration ratio between inside and outside of mitochondrial membrane is given by:

$K_{mit}=\frac{C_{mit}}{C_{aq}}=\frac{\left|HA_{m}\right|}{\left|HA_{i}\right|}=\frac{f_{n,i}*P_{n}+f_{d,i}*P_{d}*N/\left(e^{N}-1\right)}{f_{n,m}*P_{n}+f_{d,m}*P_{d}*e^{N}*N/\left(e^{N}-1\right)}$ 18 30

where

$f_{n,i}=\frac{1}{W_{i}/\gamma_{n}+K_{n,i}/\gamma_{n}+W_{i}*10^{\left(i\left(pH_{i}-pK_{a}\right)\right)}/\gamma_{d}+K_{d,i}/\gamma_{d}}$ 19

$f_{n,m}=\frac{1}{W_{m}/\gamma_{n}+K_{n,m}/\gamma_{n}+W_{m}*10^{\left(i\left(pH_{m}-pK_{a}\right)\right)}/\gamma_{d}+K_{d,m}/\gamma_{d}}$ 20

i is 1 for acids and -1 for bases, W_i_ and W_m_ are the volumetric water fraction in the cytosol and mitochondria, γ_n_ and γ_d_ the activity coefficients for the chemical in the neutral and dissociated form, pH_i_ and pH_m_ are the pH values for cytosol and mitochondria (table SM1). For the dissociated form:

$f_{d,i}=f_{n,i}*10^{\left(i\left(pH_{i}-pK_{a}\right)\right)}$ 21

$f_{d,m}=f_{n,m}*10^{\left(i\left(pH_{m}-pK_{a}\right)\right)}$ 22

For the permeability, we have for the neutral and dissociated form:

$P_{n}=\log K_{own}-0.67$ 23

$P_{d}=\log K_{owd}-0.67$ 24

where log K_own_ refers the neutral form and log K_owd_ the dissociated form. To calculate log K_owd_ from log K_own_ we use the following eq.

$\log K_{own}=\log K_{owd}+3.7$ 25

The flux of ions across the membrane is driven by the chemical and electrical potential. With the assumption of a linear potential gradient across the interface membrane, a net current flow of zero and flux in steady state (Trapp & Horobin 2005)

$N=\frac{z*mmp*F}{R*T}$ 26

where z is the electric charge (valence), mmp is the mitochondria membrane potential (volts), F is the faraday constant (96484.56 C/mol), R is the gas constant (8.314 J/mol K) and T is the absolute temperature. The values of pKa, z and I are described in table 1.

K_n,I_ , K_n,m_ ,K_d,I_ and K_d,m_ are the sorption coefficients of neutral (n) and dissociated (d) molecule for the cytosol (i) and mitochondria (m).

$K_{n,i}=L_{i}*1.22*\log K_{own}$ 27

$K_{n,m}=L_{m}*1.22*\log K_{own}$ 28

$K_{d,i}=L_{i}*1.22*\log K_{owd}$ 29

$K_{d,m}=L_{m}*1.22*\log K_{owd}$ 30

SM Table 1. Cell and mitochondrial physicochemical properties and pka, z, i for the three chemicals under study.

| **Parameter** | **Value** | **Units** | | | |
| --- | --- | --- | --- | --- | --- |
| **pH_i, cytosol_** | 7.5 | - | | | |
| **pH_m, mitochondria_** | 8.0 | - | | | |
| **W_i, cytosol_** | 0.95 | m^3^/m^3^ | | | |
| **W_m, mitochondria_** | 1 | m^3^/m^3^ | | | |
| **L_i, cytosol_** | 0.05 | g/g | | | |
| **L_m, mitochondria_** | 0 | g/g | | | |
| **γ_n, neutral_** | 1.23 | - | | | |
| **γ_d, dissociated_** | 0.74 | - | | | |
| **Chemical @ cytosol pH 7.5** | | | **pKa^a^** | **z^b^** | **i^c^** |
| Caffeine  Caffeine | | | 10.4 (40°C)  14 (25°C) | 1  1 | -1  -1 |
| Amiodarone  Amiodarone | | | 6.56*  8.8** | 0  1 | -1  -1 |
| FCCP | | | 6.2 | 0 | 1 |

a= pKa values were taken Pubchem. With exception of FCCP which was predicted by ACD labs. *O'Neil, M.J. 2001. **predicted pKa for Amiodarone using ACD labs.

The effect of a chemical in the mitochondria is given by the membrane potential change. Our proposal is to fit the experimental data mmp versus nominal chemical concentration using a two-parameter log-logistic function:

$f\left(x\right)=\frac{1}{1+\exp\left(\beta\left(\log\left(x\right)-\log\left(\alpha\right)\right)\right)}$ 31

where f(x) is the mmp and x is the chemical concentration. To fit the experimental data we use the function dmr with fct parameter set LL.2 from the R package drc.

Table SM 2. VCBA results for the three chemicals, concentration in cell (Cb), aqueous phase (Caq) and mitochondria (Cmit) for the two cell lines.

| HepaRG | | | | | | | | | |
| --- | --- | --- | --- | --- | --- | --- | --- | --- | --- |
| Caffeine | Nominal C (M) | Cb (g·gww−1) | Caq (g·gww−1) | Cmit (g·gww−1) | Caffeine | Nominal C (M) | Cb (g·gww−1) | Caq (g·gww−1) | Cmit (g·gww−1) |
| Pka 10.4 | 0.0E+00 | 2.5E-17 | 7.4E-17 | 4.5E-22 | pka 14 | 0.0E+00 | 3.4E-17 | 1.0E-16 | 1.5E-25 |
| z 1 | 2.0E-04 | 1.2E-06 | 3.7E-06 | 2.5E-11 | z 1 | 2.0E-04 | 1.2E-06 | 3.7E-06 | 6.3E-15 |
| i 1 | 3.9E-04 | 2.5E-06 | 7.4E-06 | 4.7E-11 | i -1 | 3.9E-04 | 2.5E-06 | 7.4E-06 | 1.2E-14 |
|  | 7.8E-04 | 4.9E-06 | 1.5E-05 | 9.2E-11 |  | 7.8E-04 | 4.9E-06 | 1.5E-05 | 2.3E-14 |
|  | 1.6E-03 | 9.9E-06 | 3.0E-05 | 1.7E-10 |  | 1.6E-03 | 9.9E-06 | 3.0E-05 | 4.4E-14 |
|  | 3.1E-03 | 2.0E-05 | 5.9E-05 | 2.5E-10 |  | 3.1E-03 | 2.0E-05 | 5.9E-05 | 6.4E-14 |
|  | 6.3E-03 | 4.0E-05 | 1.2E-04 | 2.4E-10 |  | 6.3E-03 | 4.0E-05 | 1.2E-04 | 6.0E-14 |
|  | 1.3E-02 | 7.9E-05 | 2.4E-04 | 2.9E-05 |  | 1.3E-02 | 7.9E-05 | 2.4E-04 | 2.9E-05 |
|  | 2.5E-02 | 1.6E-04 | 4.7E-04 | 2.8E-05 |  | 2.5E-02 | 1.6E-04 | 4.7E-04 | 2.8E-05 |
|  | 5.0E-02 | 3.2E-04 | 9.5E-04 | 1.8E-04 |  | 5.0E-02 | 3.2E-04 | 9.5E-04 | 1.8E-04 |
|  | 7.5E-02 | 4.7E-04 | 1.4E-03 | 7.4E-09 |  | 7.5E-02 | 4.7E-04 | 1.4E-03 | 1.9E-12 |
| α (IC50) | 2.2E-02 | 1.4E-04 | 4.1E-04 | 2.2E-09 |  | 2.2E-02 | 1.4E-04 | 4.1E-04 | 5.4E-13 |
| ICell Cardiomyocites | | | | | | | | | |
| Caffeine | Nominal C (M) | Cb (g·gww−1) | Caq (g·gww−1) | Cmit (g·gww−1) | Caffeine | Nominal C (M) | Cb (g·gww−1) | Caq (g·gww−1) | Cmit (g·gww−1) |
| Pka 10.4 | 0.0E+00 | 0.0E+00 | 0.0E+00 | 0.0E+00 | pka 14 | 0.0E+00 | 0.0E+00 | 0.0E+00 | 0.0E+00 |
| z 1 | 4.1E-04 | 3.0E-07 | 9.7E-07 | 4.1E-12 | z 1 | 4.1E-04 | 3.0E-07 | 9.7E-07 | 1.0E-15 |
| i 1 | 1.2E-03 | 9.1E-07 | 2.9E-06 | 1.5E-11 | i -1 | 1.2E-03 | 9.1E-07 | 2.9E-06 | 3.7E-15 |
|  | 3.7E-03 | 2.7E-06 | 8.7E-06 | 4.1E-11 |  | 3.7E-03 | 2.7E-06 | 8.7E-06 | 1.0E-14 |
|  | 1.1E-02 | 8.1E-06 | 2.6E-05 | 7.0E-11 |  | 1.1E-02 | 8.1E-06 | 2.6E-05 | 1.7E-14 |
|  | 3.3E-02 | 2.5E-05 | 7.8E-05 | 1.2E-05 |  | 3.3E-02 | 2.5E-05 | 7.8E-05 | 1.2E-05 |
|  | 1.0E-01 | 7.4E-05 | 2.4E-04 | 1.2E-09 |  | 1.0E-01 | 7.4E-05 | 2.4E-04 | 3.1E-13 |
| α (IC50) | 7.5E-02 | 5.5E-05 | 1.8E-04 | 9.2E-10 |  | 7.5E-02 | 5.5E-05 | 1.8E-04 | 2.3E-13 |
| HepaRG | | | | | | | | | |
| Amiodarone | Nominal C (M) | Cb (g·gww−1) | Caq (g·gww−1) | Cmit (g·gww−1) | Amiodarone | Nominal C (M) | Cb (g·gww−1) | Caq (g·gww−1) | Cmit (g·gww−1) |
| pka 6.56 | 0.0E+00 | 4.0E-25 | 4.8E-31 | 2.7E-31 | pka 8.8 | 0.0E+00 | 2.8E-25 | 3.4E-31 | 9.3E-34 |
| z 0 | 4.9E-08 | 1.0E-09 | 1.3E-15 | 6.9E-16 | z 1 | 4.9E-08 | 1.0E-09 | 1.3E-15 | 3.5E-18 |
| i -1 | 9.8E-08 | 2.1E-09 | 2.5E-15 | 1.4E-15 | i -1 | 9.8E-08 | 2.1E-09 | 2.5E-15 | 7.1E-18 |
|  | 2.0E-07 | 4.2E-09 | 5.0E-15 | 2.8E-15 |  | 2.0E-07 | 4.2E-09 | 5.0E-15 | 1.4E-17 |
|  | 3.9E-07 | 8.4E-09 | 1.0E-14 | 5.5E-15 |  | 3.9E-07 | 8.4E-09 | 1.0E-14 | 2.8E-17 |
|  | 7.8E-07 | 1.7E-08 | 2.0E-14 | 1.1E-14 |  | 7.8E-07 | 1.7E-08 | 2.0E-14 | 5.4E-17 |
|  | 1.6E-06 | 3.3E-08 | 4.0E-14 | 2.2E-14 |  | 1.6E-06 | 3.3E-08 | 4.0E-14 | 1.1E-16 |
|  | 3.1E-06 | 6.7E-08 | 8.0E-14 | 4.4E-14 |  | 3.1E-06 | 6.7E-08 | 8.0E-14 | 2.1E-16 |
|  | 6.3E-06 | 1.3E-07 | 1.6E-13 | 8.9E-14 |  | 6.3E-06 | 1.3E-07 | 1.6E-13 | 4.4E-16 |
|  | 1.3E-05 | 2.7E-07 | 3.2E-13 | 1.8E-13 |  | 1.3E-05 | 2.7E-07 | 3.2E-13 | 1.1E-15 |
|  | 2.5E-05 | 5.4E-07 | 6.4E-13 | 3.5E-13 |  | 2.5E-05 | 5.4E-07 | 6.4E-13 | 2.0E-15 |
|  | 5.0E-05 | 1.1E-06 | 1.3E-12 | 7.1E-13 |  | 5.0E-05 | 1.1E-06 | 1.3E-12 | 3.5E-15 |
| α (IC50) | 6.4E-04 | 2.2E-05 | 2.6E-11 | 1.4E-11 |  | 6.4E-04 | 1.0E-05 | 1.2E-11 | 3.3E-14 |
| ICell Cardiomyocites | | | | | | | | | |
| Amiodarone | Nominal C (M) | Cb (g·gww−1) | Caq (g·gww−1) | Cmit (g·gww−1) | Amiodarone | Nominal C (M) | Cb (g·gww−1) | Caq (g·gww−1) | Cmit (g·gww−1) |
| pka 6.56 | 0.0E+00 | 0.0E+00 | 0.0E+00 | 0.0E+00 | pka 8.8 | 0.0E+00 | 2.3E-23 | 2.8E-29 | 8.3E-32 |
| z 0 | 4.1E-07 | 1.0E-09 | 1.2E-15 | 6.8E-16 | z 1 | 4.1E-07 | 1.0E-09 | 1.2E-15 | 3.5E-18 |
| i -1 | 1.2E-06 | 3.0E-09 | 3.7E-15 | 2.0E-15 | i -1 | 1.2E-06 | 3.0E-09 | 3.7E-15 | 1.1E-17 |
|  | 3.7E-06 | 9.1E-09 | 1.1E-14 | 6.1E-15 |  | 3.7E-06 | 9.1E-09 | 1.1E-14 | 3.2E-17 |
|  | 1.1E-05 | 2.7E-08 | 3.3E-14 | 1.8E-14 |  | 1.1E-05 | 2.7E-08 | 3.3E-14 | 1.4E-16 |
|  | 3.3E-05 | 8.2E-08 | 9.9E-14 | 5.5E-14 |  | 3.3E-05 | 8.2E-08 | 9.9E-14 | 3.6E-14 |
|  | 1.0E-04 | 2.5E-07 | 3.0E-13 | 1.6E-13 |  | 1.0E-04 | 2.5E-07 | 3.0E-13 | 8.2E-16 |
| α (IC50) | 3.9E-05 | 9.5E-08 | 1.1E-13 | 6.3E-14 |  | 3.9E-05 | 9.6E-08 | 1.2E-13 | 3.2E-16 |
| **HepaRG** | | | | | **ICell** **Cardiomyocites** | | | | |
| FCCP | Nominal C (M) | Cb (g·gww−1) | Caq (g·gww−1) | Cmit (g·gww−1) | FCCP | Nominal C (M) | Cb (g·gww−1) | Caq (g·gww−1) | Cmit (g·gww−1) |
| pka 6.2 | 0.0E+00 | 0.0E+00 | 0.0E+00 | 0.0E+00 | pka 6.2 | 0.0E+00 | 0.0E+00 | 0.0E+00 | 0.0E+00 |
| z 0 | 5.0E-07 | 4.2E-09 | 3.5E-10 | 3.0E-10 | z 0 | 5.0E-07 | 4.9E-10 | 4.3E-11 | 3.7E-11 |
| i -1 | 1.0E-06 | 8.4E-09 | 7.0E-10 | 6.1E-10 | i -1 | 1.0E-06 | 9.7E-10 | 8.6E-11 | 7.4E-11 |
|  | 2.5E-06 | 2.1E-08 | 1.8E-09 | 1.5E-09 |  | 2.5E-06 | 2.4E-09 | 2.2E-10 | 1.9E-10 |
|  | 5.0E-06 | 4.2E-08 | 3.5E-09 | 3.0E-09 |  | 5.0E-06 | 4.9E-09 | 4.3E-10 | 3.7E-10 |
|  | 1.0E-05 | 8.4E-08 | 7.0E-09 | 6.1E-09 |  | 1.0E-05 | 9.7E-09 | 8.6E-10 | 7.4E-10 |
|  | 2.5E-05 | 2.1E-07 | 1.8E-08 | 1.5E-08 |  | 2.5E-05 | 2.4E-08 | 2.2E-09 | 1.9E-09 |
|  | 5.0E-05 | 4.2E-07 | 3.5E-08 | 3.0E-08 |  | 5.0E-05 | 4.9E-08 | 4.3E-09 | 3.7E-09 |
|  | 1.0E-04 | 8.4E-07 | 7.0E-08 | 6.1E-08 |  | 1.0E-04 | 9.7E-08 | 8.6E-09 | 7.4E-09 |
| α (IC50) | 1.9E-05 | 1.6E-07 | 1.3E-08 | 1.2E-08 |  | 1.9E-05 | 1.9E-08 | 1.6E-09 | 1.4E-09 |

**Reference**

O'Neil, M.J. (ed.). The Merck Index - An Encyclopedia of Chemicals, Drugs, and Biologicals. 13th Edition, Whitehouse Station, NJ: Merck and Co., Inc., 2001., p. 85

Trapp S., Horobin R.W. (2005). A predictive model for the selective accumulation of chemicals in tumor cells. Eur. BiophisicsJ., 959-966.

Trapp S, Rosania GR, Horobin RW, Kornhuber J. (2008) Quantitative modeling of selective lysosomal targeting for drug design. Eur Biophys J. 2008 Oct;37(8):1317-28.

Zaldívar, JM, Paini A, Joossen E, Sala Benito JV, Worth A. (present issue) Theoretical and mathematical foundation of the Virtual Cell Based Assay. Toxicology in vitro. Special issue on the VCBA.

Zaldívar Comenges JM, J. Wambaugh, R. Judson, (2012) “Modelling in vitro cell-based assays experiments: Cell population dynamics” Chapter in Models of the Ecological Hierarchy.

Zaldívar JM, Mennecozzi M, Marcelino Rodrigues R, Bouhifd M. (2010). A biology-based dynamic approach for the modelling of toxicity in cell-based assays. Part I: Fate modelling. <http://publications.jrc.ec.europa.eu/repository/bitstream/JRC58506/cell_lines_tox1stpart.pdf>

Zaldivar Comenges J, Menecozzi M, Macko P, Rodriguez R, Bouhifd M, Baraibar Fentanes J. (2011). A Biology-Based Dynamic Approach for the Modelling of Toxicity in Cell Assays: Part II: Models for Cell Population Growth and Toxicity. <http://publications.jrc.ec.europa.eu/repository/bitstream/JRC63686/lbnb24374enn.pdf>
